# TrackInk: An IoT-Enabled Real-Time Object Tracking System in Space

**DOI:** 10.3390/s22020608

**Published:** 2022-01-13

**Authors:** Cameron Aume, Keith Andrews, Shantanu Pal, Alice James, Avishkar Seth, Subhas Mukhopadhyay

**Affiliations:** 1 Faculty of Science and Engineering, School of Engineering, Macquarie University, Sydney, NSW 2109, Australia; cameron-brooks.aume@students.mq.edu.au (C.A.); keith.andrews@students.mq.edu.au (K.A.); alice.james@mq.edu.au (A.J.); avishkar.seth@mq.edu.au (A.S.); subhas.mukhopadhyay@mq.edu.au (S.M.); 2 Faculty of Science, School of Computer Science, Queensland University of Technology, Brisbane, QLD 4000, Australia

**Keywords:** Internet of Things, access control, policy management, security, architecture

## Abstract

Nowadays, there is tremendous growth in the Internet of Things (IoT) applications in our everyday lives. The proliferation of smart devices, sensors technology, and the Internet makes it possible to communicate between the digital and physical world seamlessly for distributed data collection, communication, and processing of several applications dynamically. However, it is a challenging task to monitor and track objects in real-time due to the distinct characteristics of the IoT system, e.g., scalability, mobility, and resource-limited nature of the devices. In this paper, we address the significant issue of IoT object tracking in real time. We propose a system called ‘*TrackInk*’ to demonstrate our idea. TrackInk will be capable of pointing toward and taking pictures of visible satellites in the night sky, including but not limited to the International Space Station (ISS) or the moon. Data will be collected from sensors to determine the system’s geographical location along with its 3D orientation, allowing for the system to be moved. Additionally, TrackInk will communicate with and send data to ThingSpeak for further cloud-based systems and data analysis. Our proposed system is lightweight, highly scalable, and performs efficiently in a resource-limited environment. We discuss a detailed system’s architecture and show the performance results using a real-world hardware-based experimental setup.

## 1. Introduction

**Figure 1:**
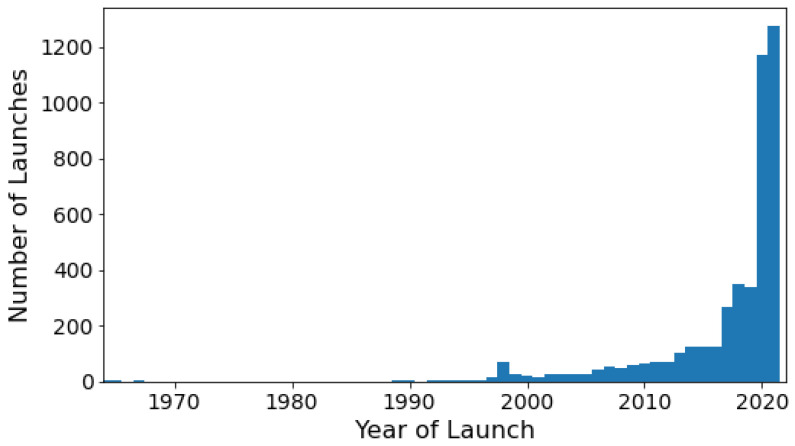
Number of launches per year for currently active satellites [28]. X-axis corresponds to the year of launch for a given satellite and Y-axis corresponds to the number of currently active satellites (units).

With the development of Information and Communication Technology (ICT), cellular networks [1], Internet of Things (IoT) applications [2,3,4], and the developments towards the space sciences, there is an increasing demand towards the innovation of satellite design, tracking systems, and their orchestrations at scale [5,6,7,8,9,10,11]. As illustrated in Figure 1, it can be seen that the number of satellites launched into orbit has increased in recent years. It highlights the demand for satellite constellations for various upfront technologies, e.g., for Global Positioning Systems (GPS) [12], global integrated terrestrial-satellite network coverage [13,14], as well as satellite internet [15,16,17]. The information provided by these satellites is useful and facilitates our daily life in many ways. For example, these satellites can provide data for real-time monitoring of weather patterns [18], GPS for navigation, advanced alarms for natural disasters [19,20], or even monitoring and predicting crops production by delivering a platform for productive, profitable, and sustainable farming [21,22,23,24,25,26,27].

On the one hand, with rapid improvements in portable sensors, wireless sensor networks, and intelligent mobile devices, there has been a huge growth in the number of devices per user in recent years [29,30,31,32,33,34,35,36]. On the other hand, there is particular interest in using these low-cost devices to perform complex calculations, raising policy enforcement and security issues in the IoT space [37,38,39,40,41,42,43,44]. Particularly for those required for orbital mechanics analysis. Traditional satellite locating devices are complex, expensive, heavy-weight, non-transportable, and generally not feasible for amateurs, leaving them mostly for high-value applications [45,46,47,48], indicating a demand for lightweight, cost-effective, and efficient tracking systems that can leverage the edge computing of IoT systems.

Existing methods for tracking satellites in orbit, among other flying objects, include passive radar signal analysis for drone and air-target detection [49,50], infrared thermal image processing for tracking hot flying objects [51], as well as visible light computer vision [52]. Another notable technology which detects aerial objects is Israel’s Iron Dome [53], which utilises active radar technology to detect ballistic missiles mid-air before launching missiles that destroy the target missile mid-air. However, our approach is to provide a simple yet complete solution that can deliver accuracy with similar systems, e.g., above.

A gap in the field of satellite tracking exists where greater portability is required. A project that requires a network of numerous small-scale interconnected devices, e.g., for complex optical satellite diagnoses or for three-dimensional (3D) imaging of satellites, a portable, low cost solution is desirable. To address these challenges, in this paper, we propose a framework called ‘*TrackInk*’—a portable, cost-effective, and efficient tracking system that uses publicly available satellite data to track satellites in space (i.e., in orbit). TrackInk aims to solve the problem of portability and cost by using lightweight consumer-grade electronics, which can easily be powered with batteries and carried by anyone. Portability will also be ensured in the system by using sensors to determine where the device is located on Earth, along with its orientation, which will be compensated for in the program workflow [54,55]. Various information from the system, e.g., geographical location and relative satellite position, will be published to an IoT analytics platform to provide remote system diagnostics.

TrackInk helps in localising and tracking the elevation and azimuth of any given satellite with public Two-line Element (TLE) data. The system framework works by propagating satellite positions from TLE sets, thus, it is easy to modify the system to track additional or new objects with available TLE data. Publicly-available TLE sets are primarily used for satellites or orbital debris. Information about other celestial bodies is also available in different forms, which can also be converted into relative positional data for use within this system. Our proposed framework has the potential to track objects including celestial bodies along with artificial satellites and orbital debris that have publicly available data, e.g., from CelesTrak [28]. This system provides the framework for additional projects relating to satellites and orbital debris. The framework described in this project could be implemented onto a ground station with a few hardware and software additions. The novelty of this framework comes from its portability, low-cost, and ability to expand for various other space-related projects. It uses a WiFi-enabled micro-controller to read online databases to retrieve public satellite data. Sensors are used to localise the apparatus to translate the reference of the satellites from a geocentric model to the reference frame of the device. This allows for the device to be placed in different orientations and different locations while still following the satellites accurately. The major contributions of this paper can be summarised as follows:

- We propose a real-time, portable, cost-effective, and highly efficient object tracking framework that is able to detect objects in space with high accuracy.
- We detail the system design and provide a complete implementation of the proposed framework with a hardware-based practical test-bed setup.
- We examine the performance of the proposed framework using a practical use-case example.

The rest of the paper is organised as follows. In Section 2, we discuss related works. In Section 3, we discuss our proposed framework for this system. In Section 4, we discuss the design and implementation of TrackInk. In Section 5, we discuss further system enhancements which could be applied to TrackInk, along with some interesting challenges we encountered during research. Finally, in Section 6, we conclude the paper with a discussion on future works.

## 2. Related Work

There are several proposals that address the issues of location tracking [56,57]. Many of them used techniques, e.g., radar-based satellite tracking, telescopic optical detection of space objects, etc. For instance, proposal [58] discusses the use of reflected radio signals transmitted by the Graves Radar to detect and track objects in LEO with the use of a low-cost directional antenna. The proposed system can validate TLE data of the ISS and detect objects with a mass over one ton. With this device relying on the Graves Radar to operate, it is geologically dependent and would need to be re-calibrated if moved.

Proposals [59,60] present the use of a camera attached to a telescope for optical detection of space objects. Both proposals achieved this by calibrating the system to a background image of the night sky, and performing computer vision algorithms to detect objects. This system is sufficient to detect objects in orbit, but is limited because it can only be run during night-time, and it also has a limited field of view compared to our proposed TrackInk system. With a similar objective to [59,60], proposal [61] discusses the use of a servo-actuated telescope to track the ISS using TLE data, visually verifying that the system is accurate. However, unlike our proposed approach, in this proposal, the telescope-servo apparatus lacks portability, both in the sense of its size and lack of environmental sensors to orient itself regardless of location.

One other article, [62], discusses the use of TLE data for satellite and planetary tracking for image capturing. This work discusses using the SGP4 algorithm to convert altitude and azimuth to actuate a telescope and camera. Computer vision is then performed to further improve the tracking of the object. The work discussed in this article requires an initial configuration in which the device must be placed level and pointed North, which is not required by our proposed TrackInk system. Another proposal discusses the use of a permanent optical ground station utilising an actuated telescope and camera apparatus [63]. This apparatus is used to perform image analysis to estimate the spatial distribution of satellites in LEO. However, unlike these systems (i.e., [62,63]), our proposal presents a simple, cost-effective, and lightweight solution for object tracking in space.

Proposal [64] discusses a decentralised approach for managing telescopes for satellite tracking. This proposal utilises the many telescopes around the world to manage and track active satellites along with other space debris. It focuses on the management of pre-existing telescopes, specifying what tasks should be done at a given time. However, it does not discuss the significant issue of portability of individual devices, which our proposed TrackInk framework provides.

In [65], the use of optical telescope observations to monitor the distance between two geosynchronous satellites, Turksat 2A and Turksat 3A, is discussed. It is discovered that an optical system is sufficient to compare the orbital paths of the two satellites and validate their simulation models. In addition, the system allows for live system analysis, which would be necessary if any perturbations caused the satellites to approach each other’s orbits. Similarly, another geosynchronous satellite, WINDS/Kizuna, is tracked in [63] using an optical telescope, using stars as a reference in space. Both of these articles utilise large permanent observatories for their sensing, which results in high costs that our proposed system avoids. Utilising shared observatories also means limited use of the telescope, which could be a detriment to the research done, which is not an issue for the low-cost implementation of our proposed TrackInk system.

## 3. Proposed Framework

In this section, we discuss the design of the proposed framework. First, we introduce the various components associated with the framework, and then provide system functionality.

### 3.1. Components

**Figure 2:**
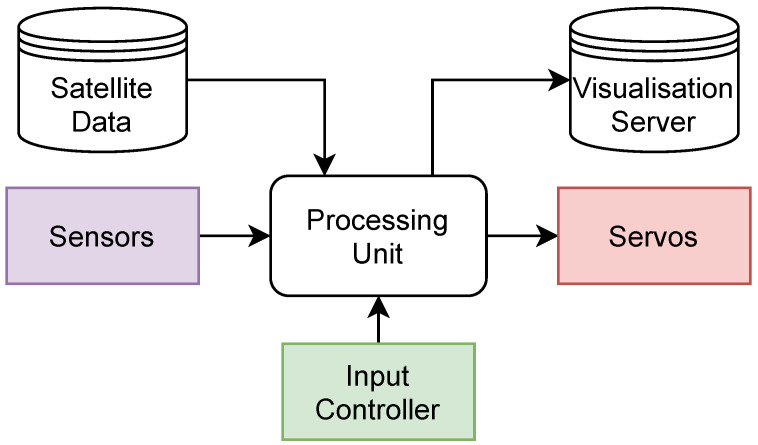
A simple outline of the proposed ‘*TrackInk*’ framework and its major components.

In Figure 2, we illustrate a simple outline of the proposed ‘*TrackInk*’ framework. It is composed of the following components: (i) Sensors, (ii) servos, (iii) input controller, (iv) processing unit, (v) satellite server, and (vi) visualisation server.

The sensors are used to collect physical parameters of the apparatus, and they help determine where the device is on Earth and its orientation. The servos are used to articulate the system and perform its desired action of indicating the location of satellites and other celestial bodies. The input controller allows user interaction with the system and determines what the device should be doing at any given time. The processing unit determines where the satellite or celestial body is relative to the sensor readings, determining which objects may be overhead, prioritising which to track, and commanding the servos to move to the desired position. The satellite server stores the corresponding satellite data that is used to determine where satellites are in orbit. Finally, the system status and satellite data are sent to a visualisation server, among other values.

### 3.2. System Functionality

Before going to the detailed implementation of the proposed framework, in this section, we discuss the system functionality of the different components discussed above.

The processing unit is the main computational device. It reads the user input, satellite data, and user input controller. It uses the user input to determine whether the system should be automated or run with a manual control. If the system is in automated control mode, it will use the satellite data and sensor data to decide which object to follow and calculate the corresponding servo positions. Information about the system, e.g., geographical location and local satellite altitude and azimuth are then sent to the visualisation server from the processing unit.

In the framework, multiple sensors are used to measure information about the system. More specifically, these sensors will be used to determine the exact geographical location of the apparatus, along with its rotation relative to Earth. These sensor readings will then be fed back to the processing unit for further use.

An input controller is used as an interface between the user and system. This will allow the user to control the state of the system, toggling between automated and manual modes. When in manual mode, the user will be able to directly control the direction of the servos. User input is ignored when the system is in automated mode, other than switching operating modes.

Satellite and celestial body data is essential for this system to work due to the fact that orbiting bodies do not follow a perfect orbit. This live data is acquired from a publicly accessible server hosting a database on TLE satellite data and celestial body data. This data is accessed and interpreted by the processing unit.

Servo motors are used to actuate the system. The purpose of this component is to move the system to point at the targeted object. A position is sent from the processing to these devices to move the system to the correct position. A visualisation server is used to visualise parameters of the system. This component is used to display plots of how the system operates and changes modes of operation over time. This both provides useful information to debug the system but is also used to show how satellite positions change over time, e.g., seeing their latitudinal sinusoidal paths due to their circular orbits.

## 4. System Development and Evaluation

In this section, first, we discuss the system implementation in detail. Then we present the achieved results to demonstrate the feasibility of the system.

### 4.1. Implementation

**Figure 3:**
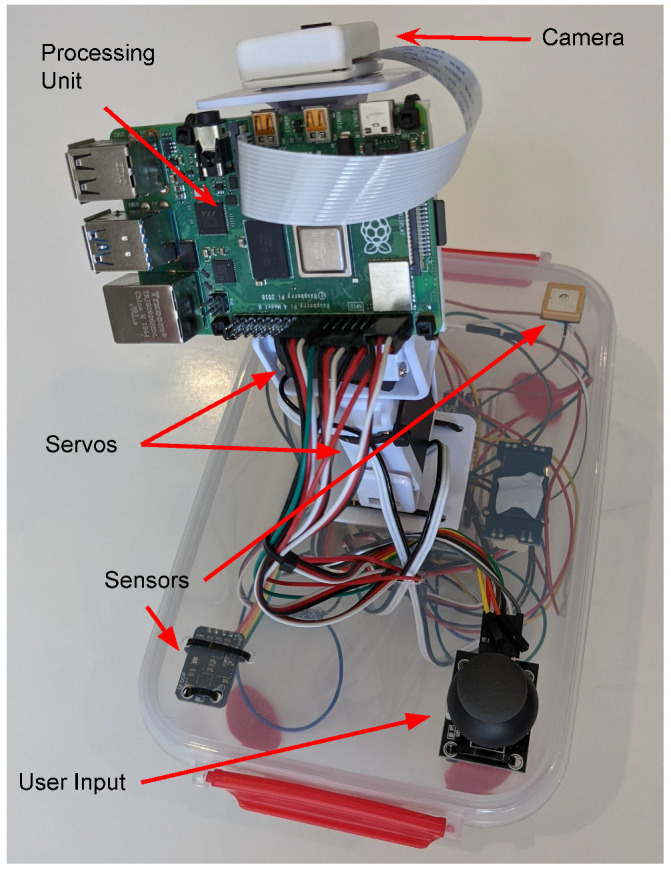
Physical implementation of the proposed ‘*TrackInk*’ framework with labelled components.

The complete hardware construction of the proposed framework is illustrated in Figure 3. The framework is implemented using a Raspberry Pi 4B+ device, 2 standard hobby servos, a Grove GPS Sensor, a DF Robot 10-DOF IMU, a 2-axis Analog Joystick, and an MCP3008 ADC Chip. These components are also connected to a LiPo battery and voltage regulator to enable it to be transported non-tethered.

The chosen processing unit device was the Raspberry Pi Model 4B+. This was chosen for many reasons, including its in-built WiFi connectivity, high processing power, vision processing capabilities, ability to plug in display for debugging, and programming purposes [66]. This device can easily interface with all of the sensors, as well as communicate with cloud services to get satellite data and publish it to the visualisation servers for further processing and display of information about the system.

**Figure 4:**
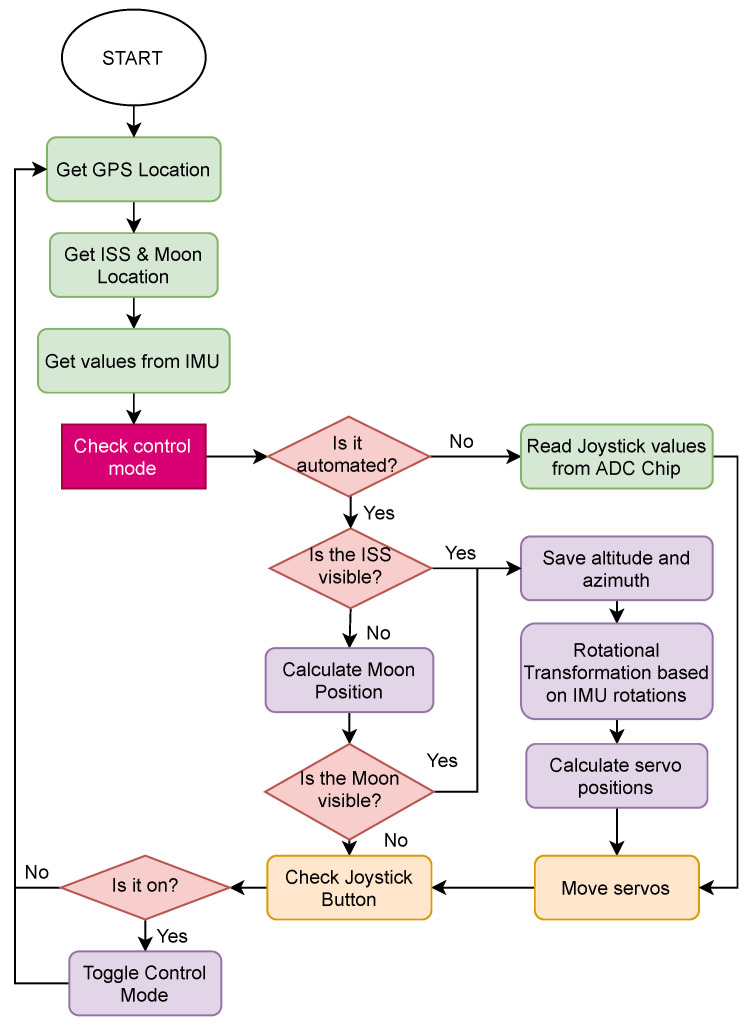
Flow of information, and interactions and communication between various components in the proposed framework.

The code was implemented in Python with class structures, allowing the corresponding python file to be placed in the working directory and imported from the required program files. Libraries were written for each device and component, allowing for individual component testing, simple system integration, and framework flexibility. A program flow of communication and interactions is depicted in Figure 4. The logical implementation of the system can be seen, where the device first gets information about its environment, decides what to do from there, then finally performs the actions, and does so continuously. The complete implementation details, along with the working codes, are available here [67].

The sensors chosen for this project were an inertial measurement unit (IMU) and a GPS receiver. The IMU contains multiple sensors, including an accelerometer, magnetometer, and gyroscope. The 3-axes of the magnetometer are used to determine which direction the magnetic field is relative to the sensor. The accelerometer is used to determine which direction is down. These two vectors are cross-multiplied to get the east direction, and magnetic North is determined by cross-multiplying east and down. These are used to determine the roll, pitch, and yaw of the system, relative to magnetic north. The IMU readings are read with I2C (Inter-Integrated Circuit) protocols and converted to the proper formats using libraries provided by the sensor manufacturer.

The IMU placement was crucial as the magnetometer is very sensitive to magnetic fields and, as such, needs to be far from unnatural magnetic sources, e.g., the servos, as possible. The orientation of the sensor relative to the servos was also compensated for by using an external compass for calibration purposes. Once the IMU was attached to the system, the relative IMU orientation was offset in the program.

The GPS receiver allows TrackInk to understand where it is located on Earth, as it requires to know where objects are relative to the device. This is used by the processing unit to perform the correct 3D transformations to accurately determine where the satellites are relative to the system.

The GPS device is communicated via a serial connection on a Raspberry Pi. The serial port is accessed where a GPS string can be read. This GPS string is in the form of an NMEA (National Marine Electronics Association) protocol and can be processed by reading the GPRMC values, which contain the time, date, position, direction of motion, and speed information [68]. This GPRMC string is processed into latitude and longitude figures by a library [69].

The input controller utilises two devices: A two-axis joystick with a button and an analog-to-digital converter (ADC). The joystick acts as the input controller, allowing the user to toggle the mode of operation by clicking the button. Once in manual mode, the user can move the servos by moving the joystick in the corresponding direction. The ADC is then used to convert the *x* and *y* values from the joystick into digital values, which can be read via SPI (serial peripheral interface).

**Figure 5:**
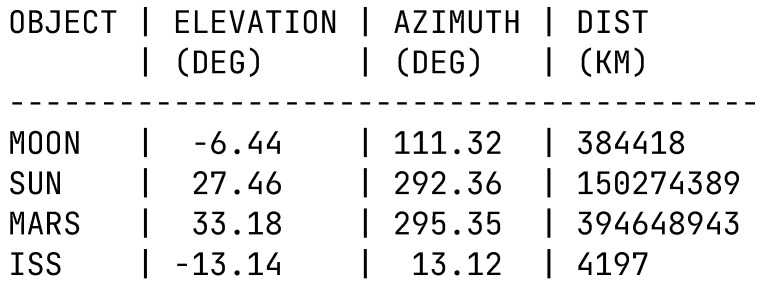
Moon, Sun, Mars, and ISS local elevations, azimuths, and distances. The elevations for the Sun and Mars are positive, meaning they were visible from the horizon at the time of measuring, while the ISS and moon were below the horizon. (DEG = Degrees).

Edge computing is utilised by TrackInk by running the main process flow within the Raspberry Pi device, converting the TLE sets into positional data which is further transformed into azimuth and elevation figures that can be sent to servo motors. The “skyfield” library is utilised to convert TLE sets into positional data and to read relative positions of other celestial bodies. This system allows for GPS coordinates (from the GPS sensor) to be input into the library to get the relative position of the given satellite or celestial body. This position is then rotated using standard rotational matrices [70] relative to the rotations from the IMU sensor readings. Additional rotations are performed to get the corresponding servo positions. An output from this program can be seen in Figure 5, detailing the values for the Moon, Sun, Mars, and the ISS.

Satellite and celestial body data were acquired from servers hosting databases. These servers are accessible via HTTPS protocols using a standard internet connection. These figures are then used by the processing unit to determine the relative position of the objects.

System information is sent to ThingSpeak via a WiFi connection for cloud-based data analysis. Each desired data point is sent to this server via an HTTPS PUT API call to be further used in graphical figures within the ThingSpeak interfaces.

### 4.2. Results

In this section, we show the results obtained from our system. The desired mode of operation for the automated system is to follow the highest priority object which, was above the horizon. Our configuration has the following priority from highest to lowest: The ISS, Moon, and Sun. This denotes that if all three of these objects were above the horizon, the ISS would be the object to be followed, and if none were visible the apparatus would stay in its last location. The Moon and Sun were chosen as objects to follow due to their frequent visibility from Earth being visible by the opened eye, while the ISS was chosen as the largest artificial satellite.

We tested our system both at night and during the day, and it was visually confirmed that the apparatus could properly point to the Moon and Sun. A time where both the Moon was visible and the ISS would fly over-head was determined, and a time-lapse video of the system was recorded. During this time-lapse, it could be seen that the Moon was rising, and with it the apparatus. After some time, the system switched to following the ISS, then moved back to the Moon. The timing of the system following the ISS was verified against NASA’s “Spot The Station” website [71].

The following figures are sent to ThingSpeak periodically: Roll, pitch, and bearing (from the IMU readings), device latitude and longitude (from the GPS sensor), satellite latitude and longitude (converted from TLE sets), and the system azimuth and elevation (to point to the satellite), and finally the control state of the system. There are only 8 fields that can be published via ThingSpeak. Thus a method of providing comma-separated values was utilised. For example, the satellite latitude and longitude were provided as a single field to ThingSpeak by combining the two values into a single string with a comma. The data was then split into the individual fields via a MATLAB script for data visualisation on Thingspeak. The main visualisations shown on ThingSpeak were the 3D orientation of TrackInk relative to magnetic north, the GPS location of TrackInk (zoomed out and zoomed in), the direction TrackInk is pointing, a control mode status indicator, and the location of the International Space Station.

**Figure 6:**
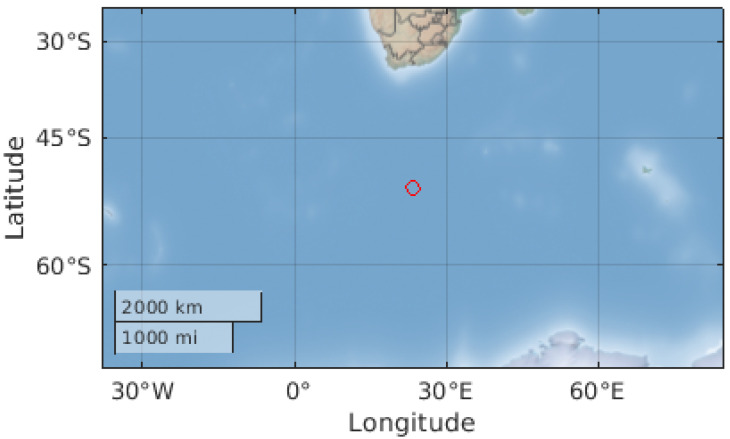
ISS location projected on Earth, just south of the tip of South Africa.

**Figure 7:**
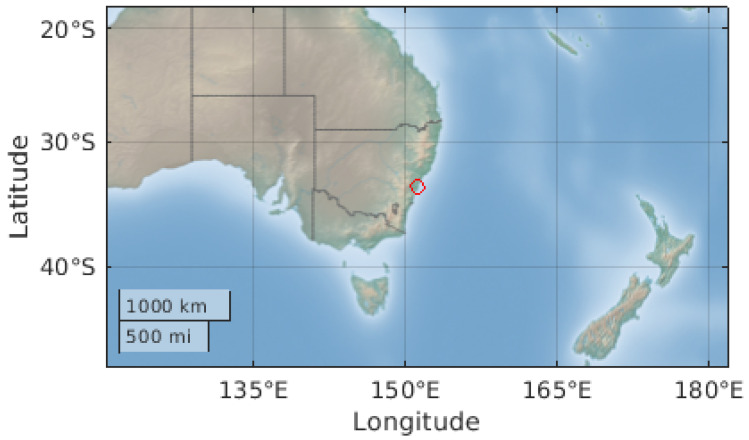
Location of the TrackInk device.

**Figure 8:**
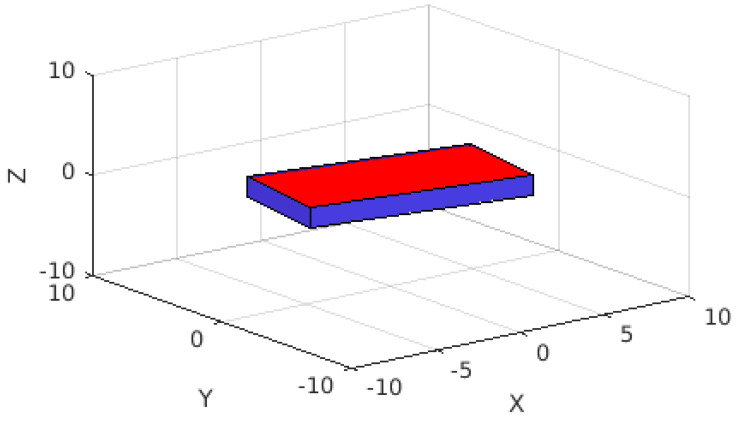
Rotation of the TrackInk device in 3D space, where the positive X axis is north and the positive Y axis is west. The blue face closest to the YZ plane represents the narrow face of the apparatus closest to the camera in Figure 3.

The location directly below the ISS can be seen in Figure 6, as displayed on the ThingSpeak server. In this figure, it can be seen between South Africa and Antarctica. Next, the location of the apparatus can be seen in Figure 7, also displayed on the ThingSpeak server. Finally, the 3D rotation of the apparatus can be seen in Figure 8, where positive X is north, positive Y is west, and positive Z is away from the Earth.

There are additional fields that contain the raw data, some of which are ‘combined’ values, e.g., the ISS GPS coordinates, which have two values in the same field separated by a comma. These combined values need to be interpreted by MATLAB to obtain the complete information encoded in them.

**Figure 9:**
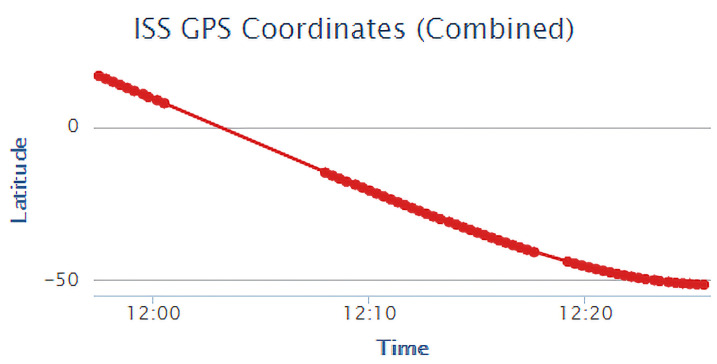
ISS Latitude over a 30-min period. Notice the expected curvature as the latitude approaches −50°.

A plot of the latitude of the ISS over time can be seen in Figure 9. In this, it can be seen that the path of travel appears linear from around 12:00 to 12:10, then begins to curve until the end of this plot. This is due to the nearly circular orbit of the ISS, in which the latitude will follow a sinusoidal curve. It can also be seen that the latitude has a minimum value of just below −50°, which is expected, since the orbital inclination of the ISS is 51.6° [72]. It should also be noted the gaps in data-points from around 12:01–12:09 and around 12:18 are from the program being restarted.

**Figure 10:**
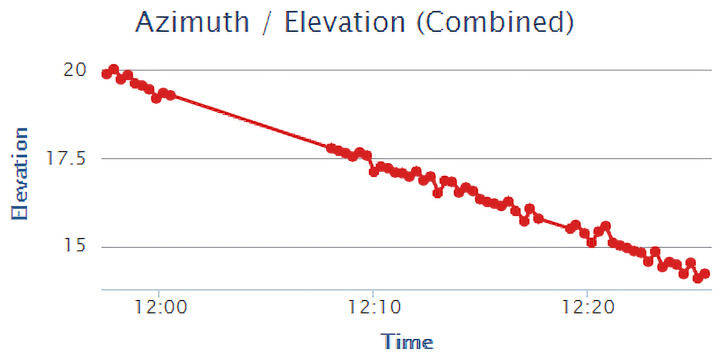
Tracked elevation of the Moon relative to TrackInk over time. A downward trend is visible as the Moon was setting at the time of recording this data. Visible noise in the data is due to variations in the IMU readings from user disturbances.

The elevation of the tracked satellite relative to the apparatus is also plotted, with the elevation of the body illustrated in Figure 10. In this plot, it can be seen that the Moon was being tracked. It can be noted that over time, the Moon is setting what appears to be a linear trend, with some slight variations, and this is due to noise from the IMU.

## 5. Lessons Learned and Discussion

Several future enhancements can be made to this system. To increase the tracking accuracy of the system, Computer Vision (CV) can be used to analyse images from a camera and give feedback to the system. The OpenCV library can be used to implement this feedback loop by detecting where the tracked object is within the camera frame and adjusting the positions of the servos accordingly.

**Figure 11:**
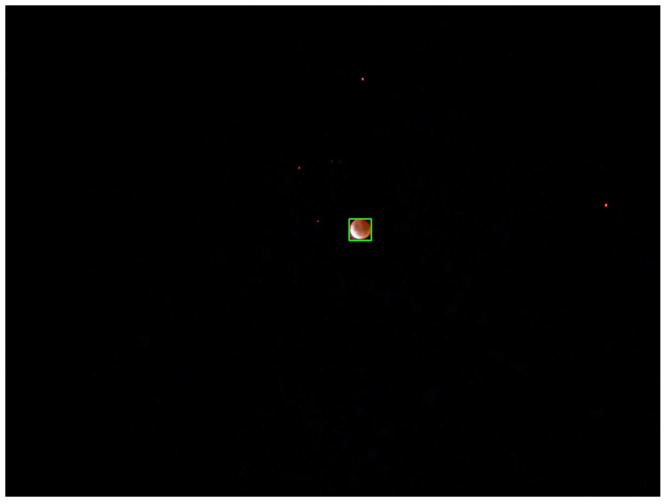
Moon computer vision tracking. Contours in the image are highlighted in red, with the largest one enclosed by a green rectangle. The centre of this rectangle can then be further used as the centre of the Moon.

This system can function by taking the gray-scale picture from the camera and creating a mask for all pixels above a specified brightness, filtering out only the brightest pixels in the camera’s field of view. Contours can be taken from this mask, and the largest object can be determined, which can be assumed to be the object that should be tracked. A bounding rectangle can be utilised to determine the centre of the object. This centre offset value can then be utilised as an offset which will be sent to the servos. It should be noted that this specific functionality would only work at night, while the system as a whole is able to function during the day. A picture of the Moon with the described CV algorithm can be seen in Figure 11, where the red line is the contour of the Moon, and the green box is the Moon’s bounding rectangle.

There are further additional improvements with regard to the IMU sensor implementation. The gyroscope had issues with regards to drifting over time, while the magnetometer was very sensitive to environmental interference. In order to avoid these issues, a sensor fusion algorithm, e.g., a Kalman filter [73] could be used to further improve reliability and avoid any individual sensor’s shortcomings.

As system portability is important, a 2-cell Lithium-Ion battery was connected to the device with a voltage regulator. This allowed for a small-sized system to power the device. Additional energy harvesting systems could be implemented, e.g., the use of photo-voltaic panels, but were not implemented into this system due to their additional complexity.

Note, the number of channels of data channels is limited to 8 on a free account on ThingSpeak. Our implementation of the project required that 10 channels of data would be uploaded (roll, pitch, yaw, latitude, and longitude of the device, elevation and azimuth of the tracked object, latitude and longitude of the ISS, and automated state of the device). This meant that some of the data must be combined into one field. This was done by separating data by commas in a given field. For example, the latitude and longitude coordinates of the ISS were joined into one channel, and later split into their given coordinates via a MATLAB visualisation. If further expansion and commercialisation of this project were done at scale, a paid ThingSpeak license would be acquired.

**Figure 12:**
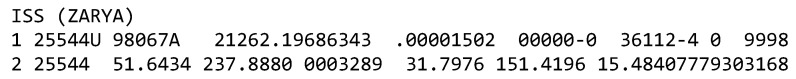
ISS two-line element data. This contains information e.g., the satellite catalog number, international designators, and the first and second derivatives of the mean motion, allowing for derivation of position, velocity, and acceleration.

The common format of satellite orbit was found in the format of a two line element set (TLE), containing an object’s orbit, motion, and time data [28] and illustrated in Figure 12. CelesTrak provides updated TLE data infrequently, around once every 6 hours. The python library utilised propagates the old TLE using simplified perturbations models (SGP4) to get the current orbital information for a given satellite.

The system described throughout this paper allows for a unique and flexible framework for applications in the space and sensor areas of research. Other similar systems have significantly higher cost and lack any portability [63,65]. Despite this, the described framework is able to provide a qualitative analysis of following and indicating toward satellites and celestial bodies in the sky.

One limitation within TrackInk is the tracking precision. Several items in the system, including the servos and TLE data cause slight misalignments when pointing to the satellites. The servo accuracy could be improved by replacing the hobby servo motors with stepper motors, which can move with higher precision. Any issues with the TLE data being inaccurate could be addressed with the use of the previously mentioned computer vision systems, allowing for a tuneable feedback loop.

## 6. Conclusions and Future Work

In this paper, we proposed a low-cost, portable, and IoT-enabled real-time satellite tracking system called TrackInk. The name TrackInk, derives from the observation that the system can efficiently track (Track) and then point out the exact location of the object in space (Ink). Our proposed approach takes advantage of the GPS. The employed sensors contain GPS chips capable of working by sending coordinates accurately. We designed and developed this system to utilise hobby-grade materials with full implementation. That said, we provide a complete set of hardware-based performances for the system. We also provide a detailed discussion on the achieved results by a real-time object tracking example. In future, we intend to implement a wireless system control feature from ThingSpeak, such that the user is able to change the functionality of the system without interacting with the physical device. The current implementation requires the user to press a physical button to change the automation status, and also requires changing code to modify the priority of tracked objects.

Additionally, future work could be done by creating a network of devices in multiple locations which could view a satellite from multiple angles. This would allow for increased viewing times of a satellite, along with the option to view a satellite from multiple angles at the same time. Higher quality cameras could also be implemented in order to give sufficient system feedback for precise movement. The issue of security in such portable devices also needs to be considered, along with data privacy concerns. Nevertheless, this is a separate direction of research that we leave for future work.

## Data Availability

Not applicable.
